# Partial Discharge and Internet of Things: A Switchgear Cell Maintenance Application Using Microclimate Sensors

**DOI:** 10.3390/s21248372

**Published:** 2021-12-15

**Authors:** Radu Fechet, Adrian I. Petrariu, Adrian Graur

**Affiliations:** 1 Computers, Electronics and Automation Department, Stefan cel Mare University of Suceava, 720229 Suceava, Romania; radu.fechet@usm.ro (R.F.); adrian.graur@usv.ro (A.G.); 2 MANSiD Research Center, Stefan cel Mare University of Suceava, 720229 Suceava, Romania

**Keywords:** electrical grid, switchgear, preventive maintenance, IoT, partial discharge

## Abstract

This paper proposes a solution for the development of microclimate monitoring for Low Voltage/High Voltage switchgear using the PRTG Internet of Things (IoT) platform. This IoT-based real time monitoring system can enable predictive maintenance to reduce the risk of electrical station malfunctions due to unfavorable environmental conditions. The combination of humidity and dust can lead to unplanned electrical discharges along the isolators inside a low or medium voltage electric table. If no predictive measures are taken, the situation may deteriorate and lead to significant damage inside and outside the switchgear cell. Thus, the mentioned situation can lead to unprogrammed maintenance interventions that can conduct to the change of the entire affected switchgear cell. Using a low-cost and efficient system, the climate conditions inside and outside the switchgear are monitored and transmitted remotely to a monitoring center. From the results obtained using a 365-day time interval, we can conclude that the proposed system is integrated successfully in the switchgear maintaining process, having as result the reduction of maintenance costs.

## 1. Introduction

**Figure 1:**
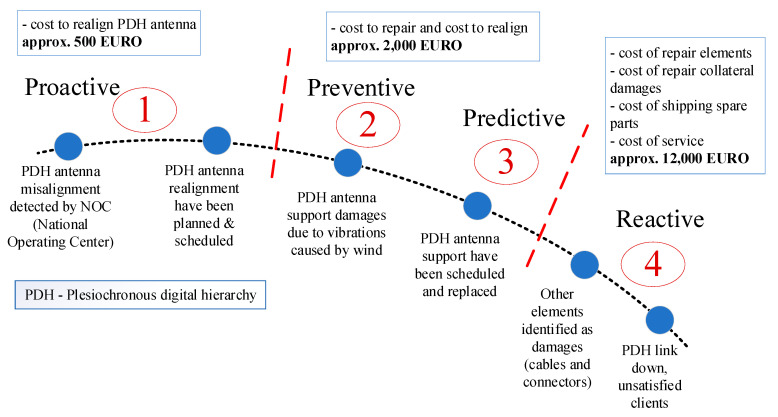
Example of maintenance costs in telecommunications and potential-to-failure curve.

Preventive maintenance and predictive maintenance are designed to reduce the amount of reactivity to failures and to increase the reliability of assets, both being a form of scheduled maintenance. As is presented in Figure 1, there are four types of maintenance depending on the execution time: proactive (the shortest and the cheapest), preventive, predictive and reactive (the longest and the most expensive). These estimations from Figure 1 are made by a real-life experience of the authors in the telecommunication domain and can be extended for all domains that involve using the equipment and hardware infrastructure.

Preventive maintenance is based on work orders that are well scheduled before maintenance is performed. This type of maintenance can be compared to an annual or bi-annual physical check, to prevent future breakdowns or emergency maintenance issues and extend the functional life of assets. One of the main challenges with preventive maintenance is balancing the cost with returns. Thus, at this point, an experienced maintenance manager is needed to make smart decisions regarding which devices require preventive maintenance and how frequently it is needed.

Predictive maintenance is scheduled based on the actual equipment condition or asset conditions and reduces material costs and labor. The main difference between the two is that predictive maintenance is scheduled as needed, based on asset conditions while preventive maintenance is scheduled at fixed intervals, in a regular way. Implementing a predictive maintenance program requires resources such as money and training. These costs are often acceptable to companies that have already successfully implemented a predictive maintenance program. Presently, many companies are on the right part of the potential-to-failure curve, doing reactive work, meanwhile, some companies are doing predictive maintenance to reach proactive maintenance. According to Figure 1, the reactive domain has negative effects as increasing the maintenance costs, causing unscheduled downtimes, forcing the company to perform unplanned work, and spending higher costs on shipping spare parts.

Generally, for implementing predictive maintenance some of the main components are necessary such as: early fault detection, fault detection, time to failure prediction, data collection and preprocessing, resource optimization and maintenance scheduling. Predictive maintenance has also been one of the driving forces for improving productivity [1].

In evaluating equipment condition, predictive maintenance uses non-destructive testing technologies such as: sound level measurements, vibration, infrared, acoustic (partial discharge and airborne ultrasonic) or oil analysis, corona detection, inside camera monitoring and other specific online tests. In most of the applications, the site measurements are supported by wireless sensor networks (WSN’s) to reduce the wiring cost and for easy configuration of the measurement setup [2].

In collaborative process automation systems (CPAS) there are performed two types of measurements: one on the actual equipment and one involving process performance measured with help of other devices. Thus, equipment maintenance is triggered. Predictive maintenance can evaluate the condition of equipment by performing continuous (online) or periodic (offline) equipment conditions monitoring. Remote monitoring by collecting data continuously can help preventive care and can also diagnose failures earlier using Internet of Things (IoT) technologies. When the Low Voltage or Medium Voltage switchgear cells (LV/MV) are involved, for example, permanent monitoring of the environmental conditions is required. These aspects are needed to prevent electrical failures due to partial discharges (PD) and extend the asset lifetime. These environmental conditions may be temperature, humidity, dust, and ozone, aside from the PD sensor. Predicting the remaining useful life of an asset using supervised machine learning (ML) is the most common technique in predictive maintenance as is mentioned by the scientific researchers in [3,4].

In this paper, we will present the concept design, development and implementation of a low-cost, open-source monitoring system based on the Internet of Things (IoT) architecture. The proposed system implies minimum system requirements and we attempted to use low-cost, low-power, reliable and readily available components to achieve the desired functions of a monitoring system. Analyzing the experiments performed using the proposed monitoring architecture, we show that one of the proposed systems works well by testing it with IoT-specific modules and infrastructure.

## 2. Partial Discharge (PD) in LV/MV Switchgear Cells

**Figure 2:**
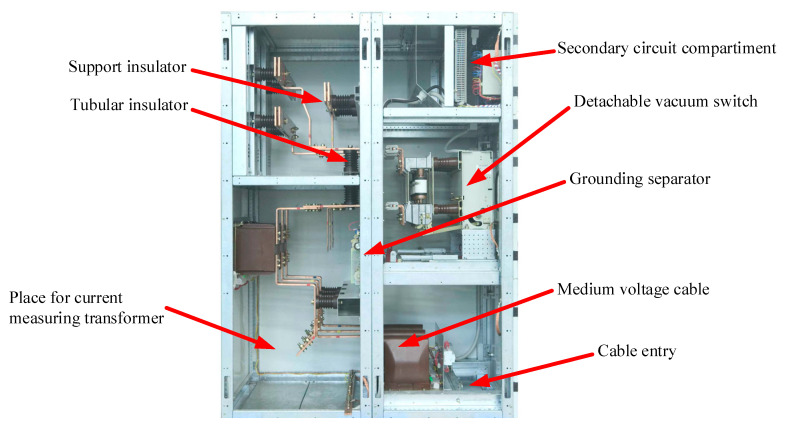
Example of MV switchgear [5].

Switchgear is an important element in an electrical grid that has a controlling and protective role, respectively. Via the switchgear, it is possible to interrupt an electrical circuit of a factory/production line or for powering a town/village, e.g., to modify/upgrade parts of the schematic circuit or to prevent further damages of the electric grid after a fault usually located downstream the grid. There are many different types of switchgears, one of them being presented in Figure 2, manufactured by ElectroAlfa, a profiled company from Romania. The main parts of a switchgear are listed in Figure 2.

In the electrical domain, partial discharge (PD) is an electrical discharge between two electrical components that does not completely bridge the space between two conducting electrodes which are at the different electrical potential in medium and high voltage equipment. The discharge may occur in a gas-filled chamber, a solid insulating material, a gas bubble, a liquid insulator, or around an electrode merged in a gas tank [6]. When partial discharge occurs in a gas environment, it is usually known as a corona [2,7].

According to IEC 60270 standard, PD is generally divided into two major sub-groups, named internal and external PD, depending on their occurrence [8]. Partial discharge (PD) occurs in gas-filled cavities or defects in the high voltage insulation and can have the following possible causes: proper manufacture failure, equipment installation, excessive use of the equipment while in service, in-service damage due to external environmental factors, aging and deterioration, particle and dust contamination [9]. PD within solid insulation system is not visible, can corrode solid insulation and eventually lead to insulation breakdown. PD can leave visible marks such as carbon tracks, discolored lines, and odors (metallic, burning or ozone). Also, a PD can cause damages that can be chemical, thermal, mechanical and emits gasses and different forms of energy such as electromagnetic or acoustic.

Due to ozone emission from PD events and because ozone is an oxidizing agent, PD is the main cause of long-term degradation and eventual failure of electrical insulation, ultimately leading to complete failure. As a result, its measurement is a standard procedure in the factory testing setup of many types of high voltage equipment. Thus, PD activity can be tested or monitored for the in-service equipment to warn against pending insulation failure.

**Figure 3:**
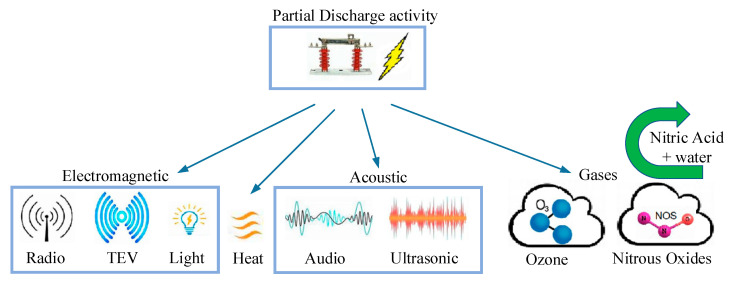
Partial discharge energy emissions.

Figure 3 depicts some of the PD energy emission types which we intend to measure in this paper using IoT infrastructure. To identify environmental conditions that facilitate PD occurrence, continuous monitoring of gases and of electromagnetic or acoustic waves is crucial [10,11]. If a PD starts, the damage to the electrical equipment will exponentially increase and could cause safety issues, the process being irreversible.

### 2.1. PD (Partial Discharge) Detection

For non-intrusive testing, the most practical techniques are based on ultrasonic and radio frequency (RF) waves detection as part of the emissions in the electromagnetic spectrum (see Figure 3).

The acoustic method for PD detection is a very attractive alternative because acoustic signals from a PD source are immune to electromagnetic noises. Because the switchgear is grounded, acoustic sensors can be positioned in a safe way at any point of the switchgear wall to detect the acoustic emission of any PD that can occur. Acoustic and ultrasonic sensors with a frequency band range from 10 kHz to 300 kHz, like condenser microphones, sound-resonance sensors, accelerometers, or piezoelectric transducers, could detect an acoustic signal from mechanical vibration of any PD. When a partial discharge is approaching equipment failure, the emission frequency decreases sometimes to the audible range. Another strong point of the acoustic method is that the acoustic signal has a dependence upon the geometry of the DUT (Device Under Test). Thus, the acoustic method is very used in the localization of PD sources inside switchgear because of its immunity against electromagnetic noise. There are also two problems: the acoustic wave propagation and the required sensitivity of the acoustic signal (very low intensity), so, to detect PD, sensors must be sensitive to small changes in amplitude of the measured signal. Using the airborne ultrasonic microphone is a sensitive method of air path of PD detection. Usually, PD detection devices using ultrasonic sensors convert emissions into audible signals so they can hear the PD activity.

PD activity which causes Transient Earth Voltages (TEV) was first discovered by Dr. John Reeves in the 1970s at EA Technology. Dr. John Reeves concluded that PD activity and TEV signals have a direct dependence and could be an indication of the status of switchgear insulation. If the switchgear is in service, PD activity may be detected by placing sensors on the outside of the switchgear.

Ultra-High Frequency (UHF) PD detection was mostly oriented to gas insulation substation (GIS) at the beginning. After years of research, ultra-high frequency partial discharge detection can be used also in non-gas insulation electrical power devices such as transformers, cables, switchgears, etc.

PD detection using optical sensors has a dependence on different factors such as temperature, pressure, PD intensity and insulation material. Mainly there are two techniques of optical PD detection, surface detection and opto-acoustic measurement. The main advantage of the last method is high sensitivity and immunity from electromagnetic interferences.

### 2.2. PD (Partial Discharge) Measurement

Switchgear PD activity can be identified and saved using several methods, all depending on the sensor technologies used. Below is a brief presentation.

- Ultrasonic microphone with a center frequency of 40 kHz. Measuring devices are available to convert PD ultrasonic emissions into audible signals, so they can detect changes in PD activity. Data from ultrasonic sensors can also be displayed and recorded locally as numerical decibel values.
- Transient Earth Voltage (TEV) sensor or coupling capacitor, from 3 MHz to 100 MHz. In this case, TEV signals are measured, displayed, and locally recorded using the appropriate instrument. The sensitivity is not likely to be sufficient to detect issues within solid dielectric cable systems.
- Ultra-High Frequency Sensor (UHF), with a detection bandwidth between 300 MHz and 1.5 GHz. UHF radio emissions are also measured, displayed, and locally saved on a dedicated device. More sensitive measurements, particularly at higher voltages, can be achieved using built-in UHF antennas or external antennas mounted on insulating spacers in the surrounding metalwork.
- High Frequency Current Transformer (HFCT) with a bandwidth between 500 kHz to 50 MHz. This method is ideal for detecting and determining the severity of the PD by burst interval measurement. The closer the bursts get to “zero voltage crossing” the more severe and critical the PD fault is.
- Acoustic Contact Sensor with detection bandwidth between 20 kHz to 300 kHz.
- Phase-resolved analysis system, which compares pulse timing to AC frequency.
- New studies present another method to detect PD by measuring the O_3_ and NO_x_ gases [12,13], which in our opinion would be a cheaper solution compared to the above-mentioned solutions.

The theory reported in [14,15] explains why corona and surface discharges have higher frequency spectra compared to internal PD. Therefore, frequency spectra of the surface discharges are higher than the frequency spectra of the internal PDs.

### 2.3. PD (Partial Discharge) Monitoring

To detect PD activity in switchgear we need continuous monitoring of the cell. Thus, the PD activity must be recorded and the same sensor technologies as Partial Discharge measurements are used for data acquisition. The sensors will monitor:

- Changes in Partial Discharge activity over time.
- Changes in Partial Discharge activity related to environmental conditions, including vibrations, temperature, and humidity.

**Figure 4:**
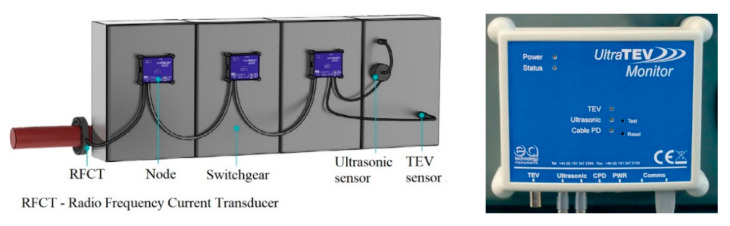
System layout of an UltraTEV Monitor System and an UltraTEV node [16].

To record and analyze changes in PD activity over a dedicated period of time, mobile PD monitoring systems are typically used, and an example is the one from EA Technology, named UltraTEV Monitor (Figure 4).

These portable devices include multiple sensors which can be mounted on various types of assets and can be linked to several central server units for data storage and remote access. Thus, PD data can be collected and analyzed in online or offline mode. This type of monitoring system is often used to provide a status regarding the health of the assets which have historical events and/or where ongoing reliability is a critical issue.

### 2.4. Ozone Measurements in PD (Partial Discharge) and Literature Overview

Ozone (O_3_) is a highly reactive gas consisting of three oxygen atoms with an unstable temperature-dependent connection between atoms. Its half-life in the air is of 3 days at +20 °C and can go to 3 months at −50 °C. Inside buildings, the indoor O_3_ half-life is usually less than 30 min and its typical value is between 5 to 20 ppb (parts per billion). This gas can be a natural product in the stratosphere (O_3_ layer being at 15 km to 45 km above sea level) or can be man-made in the troposphere (ground level).

The stratospheric O_3_ is formed naturally by the interaction of the sun’s ultraviolet rays (UV) with oxygen (O_2_). The O_3_ layer reduces the volume of harmful solar UV radiation on the Earth’s surface like a shield.

O_3_ at the ground level can be found also naturally in small concentrations of 20 to 35 ppb or because of human activity when is formed from photochemical reactions (UV) on polluted air with two major pollutants, nitrogen oxides (NO_x_) and volatile organic compounds (VOC). Nitrogen oxides can result primarily from combustion at high temperatures from industrial boilers and furnaces, electrical powerplants and combustion motor vehicles. Within the last decade, higher ambient O_3_ concentrations have been observed in the presence of sunlight and heat in summer months, and in cold months, when temperatures are below or near freezing point and snow is on the ground. O_3_ contributes to the urban “smog” which manifests especially in the summertime and sometimes in mountain regions, and it is an important element in the measurement of the Air Quality Index (AQI).

When external PD occurs in electric LV/HV switchgear cells, the chemical reaction can produce O_3_, because electrical discharges usually have sufficient power to convert O_2_ from the air into O_3_. Following the mentioned, the switchgear could be a potential source of O_3_ and can be an indirect measurement parameter to prove the PD presence. Because O_3_ is a powerful oxidizing gas, its reaction with oxidizable materials such as polymeric or ferrous metal leads to their consumption and decrease of O_3_ concentration. In the air, O_3_ reactions with other contaminants also consume O_3_ through oxidation and environmental conditions such as temperature and moisture can facilitate these reactions [17].

Many analyses were made in the scientific literature concerning the PD phenomena and its association with environmental conditions, especially air humidity. However, few examples of quantitatively predicted breakdown voltage have been reported.

In [18], this aspect is investigated using some measurements and predictions upon the obtained results. Their experiment implies using a commercial ion counter placed in a thermostatic chamber capable of controlling temperature and humidity and they evaluate negative-ion density quantitatively reported to the humidity level. According to [18], when the humidity in the air increases, the oxygen molecules changes to a clustered ion structure, forming water drops that can easily attach to a conductive layer. Testing this aspect in the PD environment, the authors obtained a negative-ion density of 400 cm^−3^ at 150 mm between a needle and a metallic plate for an RH of 10% and the value will increase up to 800 cm^−3^ for the same electrode distance when the RH is 90%.

In [19], the PD severity is analyzed by detecting the decomposition components in air-insulated switchgears under different discharge voltage and relative humidity, respectively. Thus, gases like CO resulting from the carbon contained in the needle electrode, which react with oxygen and ozone from air ionization, and NO_2_ are analyzed. For the experiments, a high voltage of 6 kV, 6.5 kV, 7 kV and 7.5 kV, respectively, are applied between a needle and a metallic plated, in a testing environment with RH values of 50%, 70% and 90%, respectively. They conclude with the severity of partial discharge divided into three levels: none or slight (CO: 0 ppm and NO_2_: 0 ppm), moderate (CO: 0–48 ppm and NO_2_: 0–30 ppm) and severe (CO: >48 ppm and NO_2_: >30 ppm). Both CO and NO_2_ in combination with H_2_O molecules will adhere to the discharge surface, acting like a corrosive agent for metal structures.

In [20], there are some related field experiments regarding the PD occurrence into switchgear. The air temperature and air humidity inside the switchgear are monitored to establish a link between them and the PD occurrence. The experiment was conducted for approximately 2 years, from July 2004 to October 2006. As the reference for the experiment, a circuit breaker energized with 11 kV was considered. Due to the microscopic cracks available in the circuit breaker insulator material, the PD frequency occurrence was high when the air humidity level was above 50%. According to the authors, the relative speed of degradation of the insulation materials (cast resin in this case) under optimum environmental conditions was remarkably rapid. The ideal approach would be to monitor the substation equipment over an extended period and record and log the environmental conditions including relative humidity and ambient temperature at the least. The same approach is in [21] where a measurement report is presented for electrical substation design and the importance of internal environmental control.

To compare the air in an LV/MV switchgear cell after PD, it is very important to analyze the initial values of O_3_ and NO_x_ in absence of the PD. It is important to note that different times of the same day have various background data for O_3_ and NO_x_, being 9.96 ppb for O_3_ and 9.1 ppb for NO_x_, respectively, according to [13]. Also, in [13], some studies were performed regarding online monitoring of PD by measuring the air chemical structure. The experiments are conducted in two air humidity level conditions (30% and 60%, respectively) with constant air temperature (25 °C) and constant air pressure, at different gap distances (15, 20, 25 and 30 mm) between a metallic needle and a metallic plate. Their results showed that after a PD, the O_3_ concentration is increased by 9–25 ppb compared with the normal value of 9.96 ppb. Also, the NO_x_ value is increased by 5 to 10 ppb compared with the nominal value measured in the normal conditions, without PD occurrence of 9.1 ppb. The values are depicted for different gap distances between the metallic needle and the metallic plate with a constant voltage applied of 4 kV between them.

The best solution is to use ultrasonic discharge counters in association with humidity and temperature sensors. New studies relate to the replacement of O_3_ sensors with ultrasonic discharge counter [22]. For example, in [22] the 40 kHz ultrasonic transducer MA40S4R from Murata Manufacturing [23], with 80° directivity, −63 dB sensitivity and 5 kHz bandwidth at −6 dB level is used for analyzing PD. Additional machine learning techniques were used to separate the parasitic surrounding noise from the PD signals.

The number of these sensor devices could be: one ultrasonic discharge sensor because it is expensive and more than one humidity and temperature sensors because they are cheap. Using a cheap integrated humidity and temperature sensor like DHT22 [24] or DHT20 [25] could be a very good solution. Another sensor like BME680 from BOSCH [26] offers integrated multiple-purpose sensors in the same chip, like air temperature, air humidity, ambient pressure, and gas concentration. BME680 reacts to most volatile compounds polluting indoor air (one exception is for instance CO_2_) and can measure the sum of VOCs/contaminants in the surrounding air (VOC = Volatile Organic Compounds). Based on an intelligent algorithm, the BSEC (Bosch Software Environmental Cluster) provides an indoor air quality (IAQ) output. In principle, this output is in an index that can have values between 0 and 500 with a resolution of 1 to indicate or quantify the quality of the air available in the surrounding area of the sensor. According to the specifications from the datasheet of the sensor, an environment IAQ index above 100 with polluting compounds could affect human health and the functionality of the equipment in that area.

**Figure 5:**
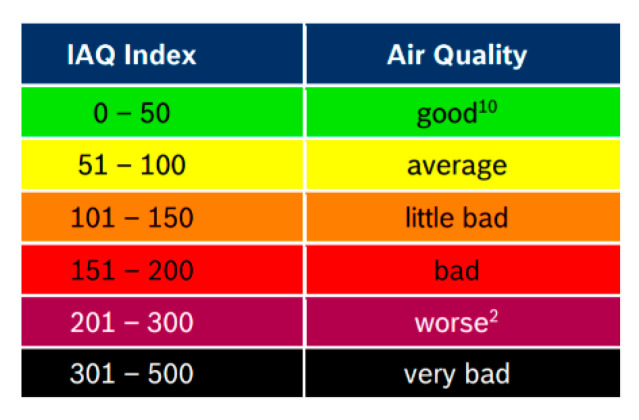
BME680 Indoor Air Quality (IAQ) and Color Coding according to the datasheet.

Dew point calculation using the above sensor type could provide good information regarding the possibility of the occurrence of PD conditions. Thus, for dew point calculation only the air temperature and air relative humidity is needed. With the help of the atmospheric pressure sensor, we can detect in advance the appearance of storms by the sudden decrease of their value. Following a storm, the humidity in the soil increases and can lead to an increase in humidity in the cable compartment of a switchgear, which can predict the appearance of a PD. Below, in Figure 5, is a color code for BOSCH’s recommended air quality, colors that we intended to use in the software monitoring for the visual identification of the air quality.

## 3. Proposed Microclimate Monitoring Architecture for LV/MV Switchgear Cell Stations

This paper presents an experimental study in the laboratory conditions of a microclimate monitoring architecture for LV/MV switchgears defined after the real-life problems meet at our local company, named ElectroAlfa. The solution is currently being tested to optimize the maintenance costs of their sold developed products.

External conditions play an important role in the performance of a switchgear. Current leaks can cause equipment to heat up. Installing a condition monitoring system with breaker diagnostics, temperature, humidity, and partial discharge systems will reduce the number of failures. The accumulated dust inside the switchgear can lead to partial discharges which can significantly damage the entire equipment. The data from sensors are received in real time—unlike preventive maintenance where the data are available only in the next maintenance interval. Loose connections can be detected through thermal sensors, faulty insulation through partial discharge sensors or faulty mechanical parts in breakers through breaker diagnostics using opening and closing time. Therefore, the risk of failure is significantly decreased with a monitoring and diagnostic solution. Remote monitoring does not make the equipment maintenance-free, rather maintenance “light” through an optimized approach, significantly decreasing maintenance and failure costs. With condition monitoring, the situation can be compared to an optimal condition as the data is available in real time from the sensors, rather than waiting for the next maintenance interval to check the condition manually and therefore, it can increase maintenance intervals by 30%.

Thus, in the case when the switchgear maintenance is dependent on the maintenance of transformers or other equipment, it is highly recommended to have condition monitoring of the whole electrification system to enhance the complete maintenance cycle.

*A*. *Annual maintenance*

According to our local company ElectroAlfa, the annual maintenance tasks are predominantly based on observation activities and a few watch ones, thus keeping the switchgear mostly powered up. Therefore, the main tasks include a partial discharge survey, exterior inspection and observation of ground contacts and checking of labels. Some annual tasks like label checking can be shifted to a three- or six-year cycle, rather than doing them every year. Partial discharge online monitoring eliminates the need to inspect the equipment with a handheld device and may predict insulation problems in advance before they become critical. Tasks like grounding inspection are still recommended by the company to be done annually.

*B*. *Three-year maintenance*

In general, each maintenance activity should be evaluated based both on the failure mode and impact on critical operations. The three-year maintenance is the most crucial activity in the complete maintenance cycle, as it is vital to justify the next six-year maintenance interval. Condition monitoring on the other hand provides data irrespective of operation topography allowing data-driven maintenance decisions. For three years of maintenance, the company has around 40 tasks that need to be completed. Remote monitoring can reduce 40 tasks to 14 tasks, providing a 65% reduction in activities, which can save money and time. Sensors’ ability to provide remotely required information is now a great advantage. Thermal and partial discharge sensors can reduce for example maintenance tasks like thermographic inspection on cable termination points which are exposed and cubicle inspection for evidence of water intrusion or other physical damage.

*C*. *Six-year maintenance (full shutdown)*

The full shutdown activities can continue as scheduled, although the intervals could be increased gradually up to 30% as online monitoring gives the critical information in real time. In the future, some full shutdown tasks might be shifted to a higher cycle (e.g., 12 years), but this should be done gradually to develop enough historical data to confirm the cycle. If sensors are working properly and giving stable data, it is not necessary to open that compartment to re-confirm after every full shutdown interval. Based on the company maintenance experience, the next two activities examples have the potential to be shifted to a 12-year plan rather than a six-year plan: check the evidence of physical damage like overheating and corona damage and inspection of all the instrument transformers for physical damages and broken leads. If it is possible (not possible at insulated busbar) it must be checked the tightness of the connections and defective wirings (vibrations can weaken the contacts over time).

**Figure 6:**
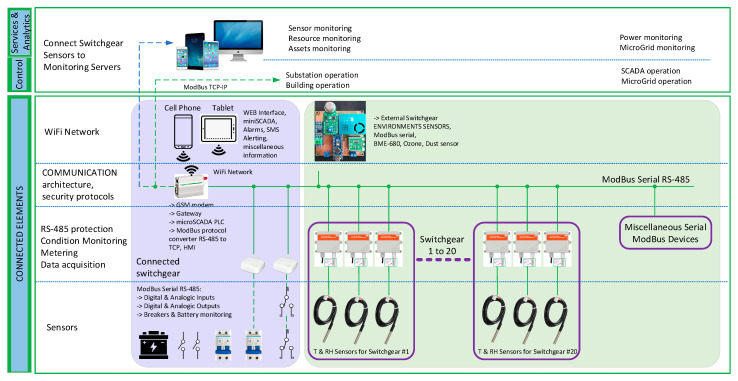
Hardware flowchart proposal for microclimate monitoring in LV/MV switchgear cells.

**Figure 7:**
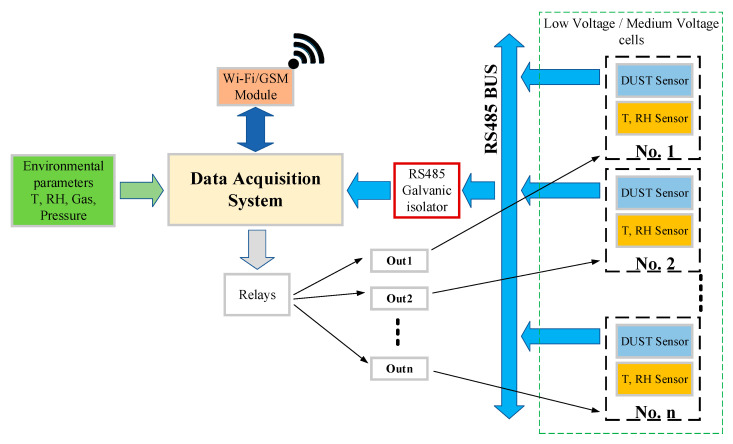
Base schematic proposal for microclimate monitoring in LV/MV cells.

To provide some data to analyze the PD discharge event in an LV/MV switchgear cell station, we use a hardware flowchart (Figure 6) and the architecture from Figure 7.

Because the proposed devices will work in harsh environmental conditions (high temperature or humidity variation, and dust appearance), the main components for this project must be characterized/tested for industrial/automotive environment, from some points of view like temperature limits of −40 °C to +100 °C and humidity from 0% to 90%. Therefore, our proposal for the hardware components is:

- Data Acquisition System (a dual band GSM router with Linux Embedded and RS-485 interface).
- DC/DC power supply (from 48 VDC–230 VDC to 12 VDC) with a backup battery with minimum autonomy of 24 h in case of the main power supply loss. The lack of power must be transmitted to the SysLog server and to a mobile terminal at the permanent maintenance service.
- Miscellaneous atmospheric and gas sensors with RS-485 interface for outside (room) and inside (LV/MV switchgear) measurements.
- GPIO’s from Data Acquisition System to ensure relay commands for power control of the miscellaneous sensors.

**Figure 8:**
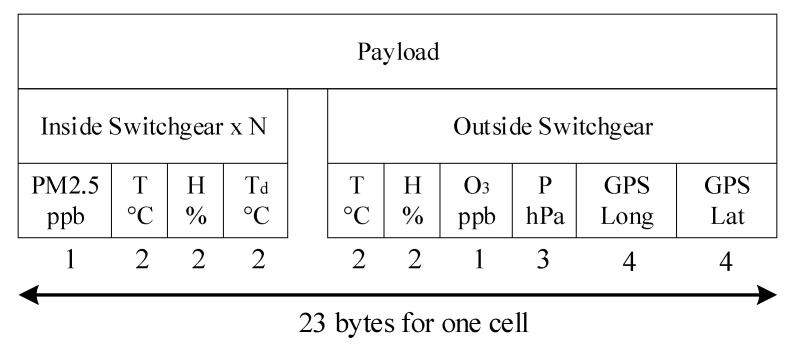
The payload needed for one cell.

Each LV/MV switchgear cell is configured in the manufacturing process with integrated sensors for dust, temperature, and humidity. Factory tests of the cell at the low voltage/high voltages are performed with the sensors inside the cell to see if they are working properly. During operation, the cell is powered up and no physical access to the sensors can be made. The GPS modules are only for the router, and they are mounted on the outside of the switchgear, to provide the exact position of the monitored distribution point. The sensors from the LV/MV switchgear cells transmit data using RS-485 BUS. Sensors are industrial, operating range is normally between −40 °C to +100 °C and their case is IP68 grade. Sensor’s response time is a maximum of 30 s, being dependent on the sensor manufacturer. Also, those sensors are integrated within a metal box, so additional shielding from electromagnetic waves is performed. Between the LV/MV switchgear cell stations and the Data Acquisition System is placed a galvanic isolator to protect the system from eventual faults that can occur with the communication lines. The role of the DAS (Data Acquisition System) is to provide enough data to be analyzed in the monitoring center. Some external sensors are mounted as well in the LV/MV cell station room to have a proper overview of the environment’s atmospheric values (temperature, humidity, gas, and air pressure). The room environmental parameters are important because they will provide functioning conditions of the switchgears and can predict eventually faults of the entire station. The communication with the monitoring center will be made through the GSM network, using GPRS (2G) or 3G/4G technologies, depending on the coverage from the area where LV/MV cells are in the field. GSM measurements need to be performed before installing the equipment, to determine if the network operators have the best coverage and the proper bandwidth for data transmission. For communication redundancy, a dual SIM GSM module modem is proposed. In case if some faults are detected, the monitoring center can transmit data to the LV/MV cell station and using some GPIO’s from the DAS and some relays, the cell can be disconnected from the grid. The total payload for the proposed architecture depends on the number of cells allocated in the same location. In case if only one cell is used, then a total of 23 bytes are needed to be transmitted at the control center (Figure 8). Furthermore, 16 bytes are for the room parameters and the location of the switchgear (being a fixed value for every monitored point) and 7 bytes are for each individual cell from the monitored point. If for example in the monitoring point are located 20 cells, then the total amount of data needed to be transmitted is 156 bytes. The system will transmit data once at 60 s, then the total amount of data transmitted per day is approximately 225 MB, which is quite low for a GSM/GPRS communication system.

**Figure 9:**
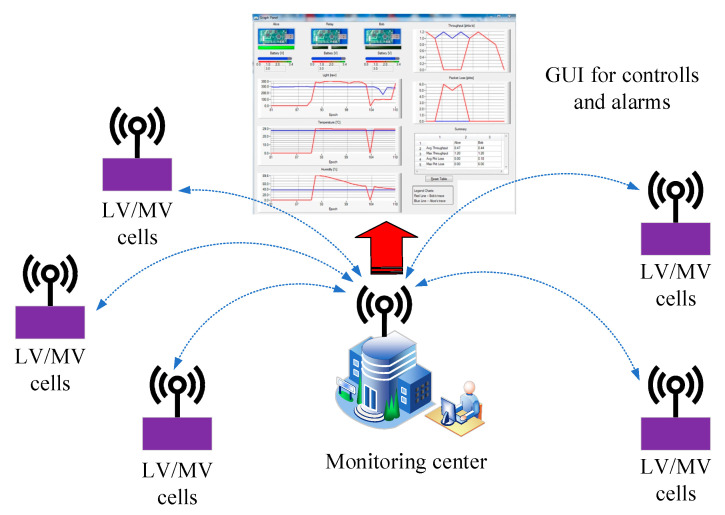
Communication flowchart proposal for microclimate monitoring in LV/MV switchgear.

The monitoring center architecture is listed in Figure 9. All the LV/MV switchgear stations from the field will transmit data and according to Figure 9, the monitoring center will analyze the received data and will display using a GUI (graphical user interface) important information about the collected parameters.

In the next section, it is briefly described data transmission between devices-server, devices-cloud platform or between IoT devices. For our scenario, only two transmission protocols are used, named ModBus and MQTT (Message Queuing Telemetry Transport).

ModBus protocol technology in our proposed architecture is used for connecting the LV/MV switchgear sensors to the acquisition system. There are currently a lot of sensors on the market using the ModBus protocol and open-source software that includes a rich palette of development languages such as: C, Java, Python, Node.js and Go.

MQTT is a lightweight publish/subscribe messaging protocol that can be used for M2M IoT (Machine to Machine) communications [27]. The main characteristics of MQTT are low power consumption, small code size, minimized data packet size, and the possibility to distribute different packets to multiple applications almost at the same time. Any network-wired or wireless device that provides a bi-directional connection can support MQTT. This MQTT protocol was specifically designed for high latency networks or low bandwidth resource-constrained devices.

The monitoring interface was designed to be available in two places: locally (SW microSCADA PLC) and remotely on more than two separate servers and using information security protocols.

### 3.1. Local Monitoring of LV/MV Switchgear

**Figure 10:**
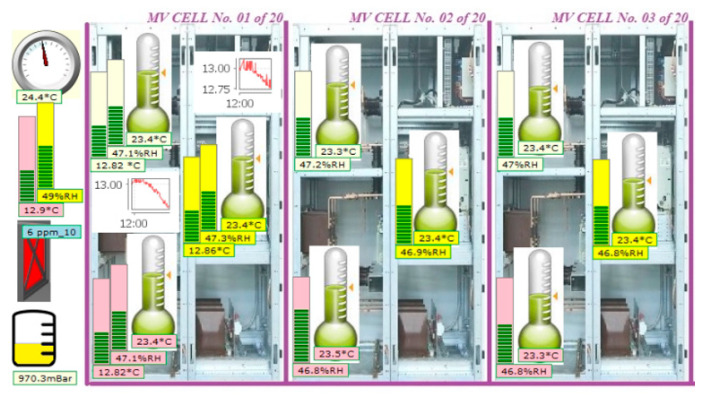
Example of locally microclimate monitoring using SCADA web page with sensors for 3 cells.

For local monitoring, a minimum SCADA will be created in DAS to observe the query of the sensors and to make a statistic for the local ModBus packages (Figure 10). In SCADA systems, to distinguish between LV/HV a general map with cells definitions is used. LV and HV cells are encoded differently by the regional electricity provider, so in the general map of LV/HV different cells will have also different names. Also, in sensors flash memory, there are some specially reserved spaces for writing a few data about the type of the equipment to which sensor belongs and equipment location (cell type and cell location in our case). The information inside the sensors can be read remotely. A more likely SCADA system web page is made on the GSM router, where are related the LV/MV cells together with the sensors. This page must be low RAM memory consumption, to not affect the GSM router OS (operational system) performances, which has only 16 MB of Flash and 128 MB of DDR2 RAM memory, 40% of the presented resources being allocated to the customized OS.

This web page is loaded from the FLASH memory, so our target is to obtain a web page less than 256 kB. With an actual unchanged web page, the actual router CPU load and RAM both stay under 30%.

Thus, with the obtained counters for serial ModBus packages, a local QoS (Quality of Service) statistic can be created that can be sent later to the server. The sizes obtained after interrogating the sensors should be stored locally for at least 7 days, which leads to the need for adequate sizing of RAM and circular buffers in the GSM router.

**Figure 11:**
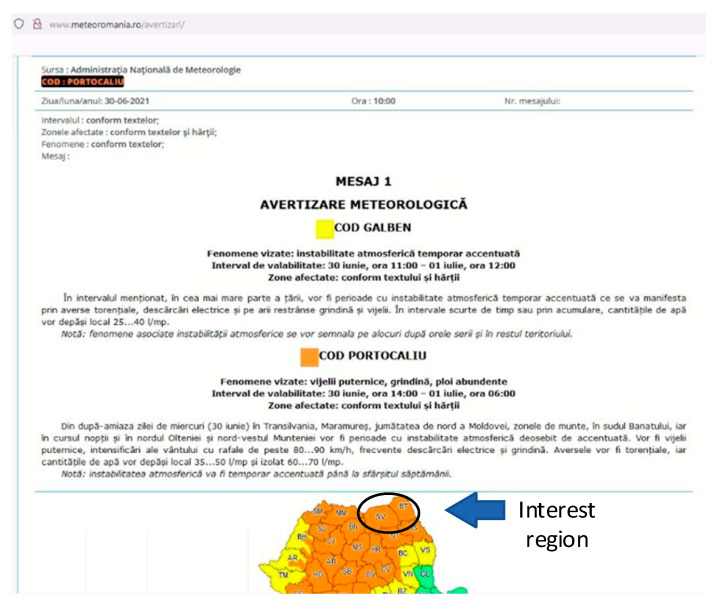
Example of Romanian weather warning, from 30 June 2021 to 1 July 2021.

**Figure 12:**
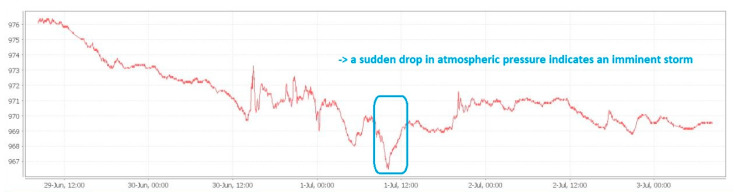
Local microclimate monitoring in LV/MV switchgear cells, indication of imminent storm: sudden drop in atmospheric pressure in 1 July 2021 before 12:00 PM.

**Figure 13:**
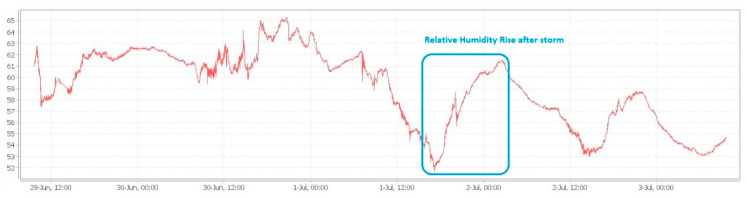
Local microclimate monitoring in LV/MV switchgear cells, increase in humidity after the storm of 1 July 2021.

An example of local monitoring (local minimum SCADA) is presented below, where the connection between the decrease of atmospheric pressure, the appearance of storms and the subsequent increase of the relative humidity in the atmosphere is observed. Our interest region, which is the North-East side of Romania (Suceava—SV and Botosani—BT) can be observed in the weather warning map, taken from our national weather forecast website (Figure 11). Thus, if we compare the timelines from Figure 11, Figure 12, and Figure 13 respectively, we can see the increasing humidity parameter measured by our system, comparing with the storm appearance in our local region.

Increasing the air humidity leads to the increase of the soil humidity also. This aspect will have the effect of increasing the humidity in the cable compartments for the LV/MV cells located in the field, especially for those located in high altitude geographic areas (mountains for example). Increased humidity in the cable compartment can lead to Corona discharge phenomena, especially on the surface of the insulators in the cable compartment. Corona discharge or PD (Partial Discharge) leads to the release of gases including ozone (O_3_) and nitrogen dioxide (NO_2_). NO_2_ gas in the presence of the water vapors and oxygen (O_2_) leads to the appearance of hydrochloric acid (HNO_3_). Both ozone (O_3_) and hydrochloric acid (HNO_3_) are oxidizing elements that can irreparably destroy over time the insulation of cables and other insulating materials in the compartments of an LV/MV cell.

An example of this measurement is performed, and the results are depicted in Figure 12 and Figure 13, respectively.

### 3.2. Remote Monitoring of LV/MV Switchgear

For the server side, the PRTG application (www.paessler.com—accessed on 5 December 2021) is currently being tested and some collected data are listed below. In the future, the Zabbix application will be also tested (www.zabbix.com—accessed on 5 December 2021) due to the advantages offered on the monitoring side for the MQTT protocol in the IoT plugin package in Zabbix Agent 2 [28] but isn’t our purpose in this paper. As a comparison between the mentioned platforms, we can say that both are generally dedicated to monitoring the IT infrastructure and can be adapted for an IoT network. There are major differences such as: the operating system used (PRTG runs only under windows/paid license, Zabbix only under linux/free license), the architecture of each application, the graphics, the configuration, software agents, etc.

The reason why the PRTG application is used in this experimental approach is that it has a considerably higher volume of accepted protocols and monitorable features than Zabbix. The design and user interface are more intuitive, modern, and easier to use. The installation and upgrade process for PRTG is also extremely fast compared to any of the Zabbix installation approaches. Another thing is that PRTG does not require agents and will take much less time to install an agent program on each remote device than in a Zabbix environment. Another plus for PRTG is that you can switch to a higher-level license by paying only the difference between the current license and the higher license fees. PRTG also offers free one-day response time from Monday to Friday, while Zabbix charges for assistance contracts and limits the number of incidents that can be called upon unless one is purchased among the top-level options. Thus, PRTG is currently the best option for switchgear network monitoring as it is an intuitive and extensive product that encompasses many communication protocols.

**Figure 14:**
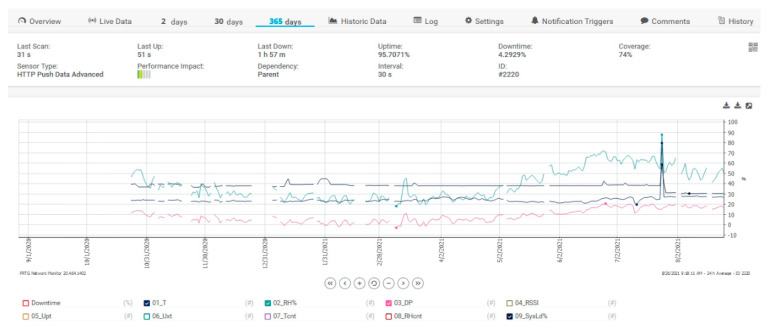
PRTG Server data for temperature, humidity, dew point and SysLoad.

**Figure 15:**
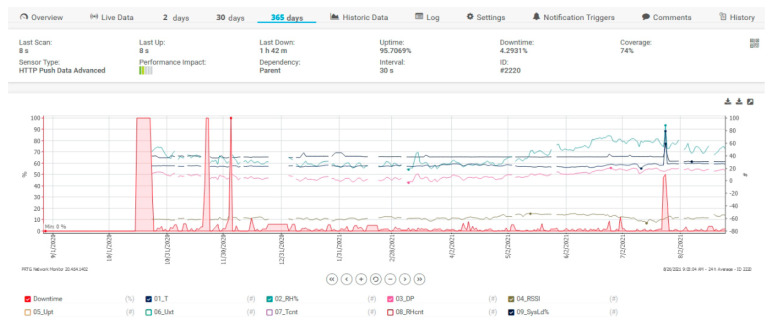
PRTG Server data for sensors and DownTime.

For collecting data, a free version of the mentioned software is used that can integrate 100 sensors only. Figure 14 shows a 365-day filter, the filter is applied for sensors with temperature, relative humidity, dew point or SysLoad for the proposed architecture. The spikes from the figure are for alarm testing purposes only and to test if everything in the proposed architecture is working. In Figure 15, some data are depicted for the same data and the same filter but is added the DownTime of the system.

**Figure 16:**
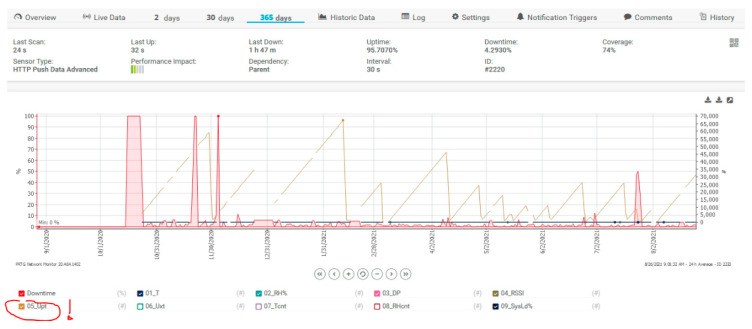
Microclimate monitoring in LV/MV switchgear using PRTG Server—365 days filter, Sensor UpTime filter checked, name 05_Upt.

DownTime intervals are for server time stability tests. The UpTime meter called 05_Upt (the red circle with exclamation mark from Figure 16) was reset for each voltage failure at the sensor, and we can see on the graph how many voltage interruptions there were at the measurement sensor. 

## 4. Conclusions

If a PD starts, the damages of the electrical equipment will exponentially increase and could cause safety issues, the process being irreversible. The LV/MV switchgear cells monitoring systems are important in optimizing the maintenance costs of the Electric Grid distribution points. Using partial discharge as a health indicator in electric power assets is somewhat like listening to heart rate or blood pressure monitoring in human health. While high levels can be useful to diagnose a problem, they need to be looked at in context and trended over time. While partial discharge analysis is not easy and is often confusing, it provides an early warning on asset failure, and as such, should be included in any comprehensive asset health monitoring program.

This paper presents a low-cost and efficient platform for IoT monitoring the LV/MV switchgear cells. The proposed architecture is defined after the real-life problems meet at ElectroAlfa company and it’s currently tested to optimize the maintenance costs of their sold developed products. The proposed monitoring system reduces the amount of time required for routine maintenance, increases the time interval of maintenance by 30% and optimizes the maintenance activities. As a result, the total cost of ownership could be reduced by up to 40%.

Through condition monitoring, the significant maintenance cost savings may reduce three-year maintenance tasks from 44 to 14 (a reduction of 65%). Not only does this save costs (e.g., labor and parts), but it also reduces the risk of damage during unnecessary inspections. Secondly, the maintenance cycle can be increased by 30%, optimizing the complete maintenance program. The decrease in operational cost and probability of failures increases the uptime. In many cases, uptime is critical in meeting operational requirements and eliminating costly downtime. There is no doubt that using effective sensors monitoring can have a positive impact on reducing costs and ensuring the smooth running of switchgear for many years to come.

## Data Availability

Not applicable.
